# Maximum tumor-absorbed dose measured by voxel-based multicompartmental dosimetry as a response predictor in yttrium-90 radiation segmentectomy for hepatocellular carcinoma

**DOI:** 10.1186/s40658-022-00520-9

**Published:** 2023-02-06

**Authors:** Javier Orcajo Rincón, Amanda Rotger Regi, Ana Matilla Peña, Laura Reguera Berenguer, Manuel González Leyte, Laura Carrión Martín, Jaime Atance García De La Santa, Miguel Echenagusia Boyra, Cristina González Ruiz, Arturo Colón Rodríguez, Juan Carlos Alonso Farto

**Affiliations:** 1 Nuclear Medicine Department, Hospital General Universitario Gregorio Marañón, Madrid, Spain; 2 Gastroenterology Department, Hospital General Universitario Gregorio Marañón, Madrid, Spain; 3 Interventional Radiology Department, Hospital General Universitario Gregorio Marañón, Madrid, Spain; 4 Dosimetry and Radioprotection Department, Hospital General Universitario Gregorio Marañón, Madrid, Spain; 5 Hepatobiliary Surgery Department, Hospital General Universitario Gregorio Marañón, Madrid, Spain

**Keywords:** Transarterial radioembolization, TARE, SIRT, Radiation segmentectomy, Voxel-based dosimetry

## Abstract

**Objective:**

Advances in hepatic radioembolization are based on a selective approach with radical intent and the use of multicompartment dosimetric analysis. The objective of this study is to assess the utility of voxel-based dosimetry in the quantification of actual absorbed doses in radiation segmentectomy procedures and to establish cutoff values predictive of response.

**Methods:**

Ambispective study in hepatocarcinoma patients treated with radiation segmentectomy. Calculated dosimetric parameters were mean tumor-absorbed dose, maximum tumor AD, minimal tumor AD in 30, 50, and 70% of tumor volume and mean AD in non-tumor liver. The actual absorbed dose (aAD) was calculated on the Y-90-PET/CT image using 3D voxel-based dosimetry software. To assess radiological response, localized mRECIST criteria were used. The objective response rate (ORR) was defined as CR or PR.

**Results:**

Twenty-four HCC patients, BCLC 0 (5), A (17) and B (2) were included. The mean yttrium-90 administered activity was 1.38 GBq in a mean angiosome volume of 206.9 cc and tumor volume 56.01 cc. The mean theoretical AD was 306.3 Gy and aAD 352 Gy. A very low concordance was observed between both parameters (rho_c 0.027). ORR at 3 and 6 m was 84.21% and 92.31%, respectively. Statistically significant relationship was observed between the maximum tumor-absorbed dose and complete radiological response at 3 m (p 0.022).

**Conclusion:**

A segmental approach with radical intention leads to response rates greater than 90%, being the tumor maximum absorbed dose the dosimetric parameter that best predicts radiological response in voxel-based dosimetry.

## Introduction

Transarterial radioembolization (TARE), also known as selective internal radiation therapy (SIRT), or simply hepatic radioembolization, is a treatment with a remarkable growth in recent years for the management of primary and secondary unresectable hepatic malignancies.

Since the beginning of the technique, progress in the knowledge of liver pathology and radioembolic therapy has brought evolving changes regarding therapeutic indications, improvements in patient selection, activity calculation models, pre and post-treatment dosimetric models, and, overall, a whole redefinition of the therapeutic objective of TARE [1, 2], leading to a more conservative approach of separate management of each hepatic lobe.

Currently, the technology available in most centers performing TARE, comprising the incorporation of cone beam CT (CBCT), SPECT/CT gamma cameras and the design of dedicated software for voxel-based multicompartment dosimetric analysis, allows for much safer planning of the procedure and the implementation of highly personalized therapies [3, 4].

Furthermore, the therapeutic approach of the technique has radically changed in the last decade [5]. TARE procedures, initially designed for the treatment of bilobar liver disease in a single procedure, have been delimiting the extent of the target volume due to different factors. The best knowledge of radiation liver damage, the obtainment of favorable results in shorter-range treatments and two-stage procedure for bilobar disease, among other factors, such as the selection of patients with a lower tumor burden and improvements in the angiographic procedure without coiling, depending on preferential flow, have led the technique to a much more sectoral approach, with higher safety margins and better oncological results [6, 7].

Under the knowledge that the safety and efficacy of TARE improve the more selective the treatment is, research into the concept of radiation segmentectomy, especially suitable for lesions smaller than 5 cm, has shed light into the suitability of this treatment for patients with small lesions confined to ≤ 2 segments [8, 9]. Results obtained in recent studies, such as the Dosisphere-01, multicenter phase II trial, or the SARAH, TARGET, and LEGACY studies, reveal the benefit of high doses of radiation to the tumor, and multicompartmental dosimetry, as determining prognostic factors [10–14].

Very recently, the new BCLC guidelines for the management of hepatocarcinoma have been published, in which hepatic radioembolization, especially that directed at small lesions and with a radical objective, is considered in the earliest stages of the disease [2]. In BCLC stage 0, defined as a solitary HCC < 2 cm without vascular invasion or extrahepatic spread in a patient with preserved liver function and no cancer-related symptoms, or in early stage (BCLC A), its implementation is now contemplated in patients who are not candidates for percutaneous ablative therapies (radiofrequency or microwave) or surgery, even in lesions up to 8 cm (based on the results of the LEGACY study) [12].

The growing interest in the influence of dosimetric and biological effects of radionuclide therapies is also one of the most important current topics, so quantitative imaging is becoming increasingly relevant in treatment planning and post-treatment dosimetric assessment workflow [15].

The dosimetric models used to know the absorbed doses on the tumor and the non-tumor liver have evolved dramatically in recent years. Initially, pretreatment dosimetric calculation methods were designed, seldom based on exact liver and tumor segmentation [16].

In order to simplify the dosimetric procedure, empirical models were designed, such as the body surface area based (BSA model), the single-compartment model (MIRD), which assumes activity is uniformly distributed throughout the source region, and the partition model, which requires complete liver and tumor segmentation, in addition to tumor-to-nontumoral liver uptake ratio (TNR) from Tc-99 m-MAA SPECT/CT. It was developed to separately estimate the absorbed dose to T, NT, and lungs [17]. This method is best used for solitary, clearly demarcated tumors [18]. However, although it is the least empirical method, it shows clear deficiencies when it comes to the exact quantification of the absorbed dose within the tumor, especially when dealing with multiple lesions, ill-defined lesions or those in which areas of necrosis and solid tissue are altered, lesions with different degrees of vascularization or with several arterial feeders amongst other situations.

Given the deficiencies of the described methods and in order to obtain a precise dosimetric study, in which lesional heterogeneity and the non-uniform distribution of MAA and microspheres are considered, voxel-based computer models have recently been designed, allowing for the calculation of the absorbed dose in each of the tissues and therefore obtaining precise and personalized dosimetry [15].

The objective of this study is to assess the utility of these new voxel-based dosimetric models in the quantification of actual absorbed doses in radiation segmentectomy procedures and to establish cutoff values predictive of radiological response to radioembolization treatment.

## Material and methods

### Study design

This single-center, ambispective study was approved by the Ethics Committee for Drug Research. 40% of the subjects were included in the study retrospectively until April 2021 and 60% prospectively from this date.

Data were obtained by searching the electronic medical record system (HCIS, Historia Clínica Digital del Sistema Nacional de Salud and Medavis-RIS Philips Healthcare).

The study inclusion criteria were as follows: (a) solitary or bifocal HCC limited to one or two liver segments, not amenable or after failure to percutaneous ablation or surgical resection, (c) absence of macroscopic vascular invasion, (d) absence of extrahepatic disease, (e) Eastern Cooperative Oncology Group (ECOG) performance status of 0–1, (f) normal baseline liver function and (g) absence of hepatopulmonary shunt that could determine an absorbed dose in the lung greater than 30 Gy.

For tumors located between two liver segments or two angiosomes (e.g., junction of segments VIII and V), the subsegmental infusion was done in each of the perfusing segments/angiosomes. If multifocal disease necessitated a lobar or more than two segments infusion, patients were excluded. Those patients in whom the calculated complete activity could not be administered or in whom the post-treatment residue was greater than 20% of the activity were also excluded from the study.

HCC was diagnosed according to the American Association for Liver Disease guidelines [19] or after liver biopsy. Patients without clinical or imaging follow-up were excluded from the analysis.

Before treatment decision, all patients were discussed in a specific multidisciplinary tumor board, comprising members from hepatology, surgical oncology, interventional radiology and nuclear medicine.

Baseline demographics for the cohort are presented in Table 1.

**Table 1:** Patients characteristics

Patient (n)	24	Range	IQR
Age (years)	63	(39–80)	56–70
Sex (%)			
Men, n (%)	19 (79%)		
Women, n (%)	5 (21%)		
BCLC, n (%)			
0	5 (21%)		
A	17 (71%)		
B	2 (8%)		
CHILD			
A			
A5	15 (63%)		
A6	5 (21%)		
B			
B7	4 (17%)		
Baseline analysis			
Br	0.9	(0.3–4.1)	0.5–1
Alb	4.01	(2.5–4.8)	3.7–4.5
ALT	37.9	(16–82)	25–49
AST	38.3	(20–50)	31–47

*BCLC*: Barcelona clinic liver cancer; *Br*: bilirubin; *Alb*: albumin; *ALT*: alanine aminotransferase; *AST*: aspartate aminotransferase; *IQR*: interquartile range

**Table 2:** Treatment characteristics

		Range	IQR
Microsphere type			
Glass, n (%)	21 (87.5)		
Resin, n (%)	3 (12.5)		
HPS, %	9,12	3.3–19	5.26–11.65
Target volume, mL	206.9	25–700	90–243.6
Administered activity, GBq	1.39	0.19–5.8	0.545–1.615
Theoretical tumor dose, Gy	306.3	250–500	300–300
Confirmed tumor dose (aAD) Gy	352	127.4–1171	168.9–462.7
Maximum confirmed dose, Gy	1216	439.1–2767	779.8–1378
Min dose in 30% of tumor volume	398.9	158.4–1295	183.1–523.5
Min dose in 50% of tumor volume	303.5	97.4–1138	138.5–384.2
Min dose in 70% of tumor volume	205.9	43.5–1011	107.9–303.9
Healthy liver dose	11.64	0.77–39.19	4.15–15.8

*HPS*: hepatopulmonary shunt; *mL*: milliliter; *GBq*: gigabecquerel; *Gy*: gray; *IQR*: interquartile range

### Technical aspects

Procedures were performed by 5 interventional radiologists and 3 nuclear medicine specialists in a center with more than 15 years of experience in this particular procedure.

Each radioembolization treatment included a mapping angiogram, performing cone beam CT. The albumin macroaggregates labeled with Tc-99 m administration were carried out slowly and in bolus, with a minimum volume of 6 ml. Thoracoabdominal planar images and SPECT/CT images of the abdomen were subsequently acquired no longer than 1 h from the start of the infusion time. The lung shunt fraction was determined.

Y-90 glass microsphere (TheraSphere; Boston Scientific Corporation) or Y-90 resin microsphere (Sir-spheres, Sirtex Medical) infusion was performed according to the standards required by manufacturers. Post-therapy Y-90-PET/CT acquisition was performed within the following 2 h from the infusion. PET/CT studies were performed on a Discovery MI PET/CT equipment, GE Medical Systems with CT parameters: Helical Full 0.5 s, thickness 3.75 mm, 120 kV, caredose, and 10-min acquisition per bed, QCFX-S3000 reconstruction.

Finally, post-treatment dosimetric analysis was performed on the Y-90-PET/CT image using the 3D voxel-based dosimetry software, MIM SurePlan LiverY90—MIM.

### Activity calculation

In all the segmental treatments ablative‐level dosimetry was used with the intent to cause complete tissue necrosis of the treated area (radiation segmentectomy). The theoretical target radiation dose was 250–400 Gy (based on MIRD—Medical Internal Radiation Dosimetry for glass microspheres and partition model for resin microspheres) in which tumor/healthy liver uptake ratio is also taken into account. Different absorbed dose targets were applied given the rapid evolution of dosimetric knowledge. In recent years, the recommended target absorbed dose for radiation segmentectomies by the most recognized working groups has gone from 190 Gy (Vouche et al. HEPATOLOGY 2014;60:192–201), going through the 250–300 Gy recommended after the Dosisphere-01 study, up to 400 Gy recommended by studies such as LEGACY. For this reason, and due to the partially retrospective nature of our study, we had to include radiation segmentectomies treated with different dosimetric objectives.

The volume used to calculate the activity (angiosome volume) was determined by volumetric software on the enhancement areas in the cone‐beam computed tomography (CBCT).

Actual administered activity corresponded to the initially calculated activity for the treatment minus the post-treatment residual activity. The theoretical absorbed dose (tAD) was defined as that calculated using the activity/dose calculation model or partition model.

### Dosimetric study

The dosimetric calculation was carried out through Workflow Y90 LDM Dose Calculation for Y-90-PET/CT image and dose–volume histogram and isodose curves.

Liver and tumor segmentation was carried out using Non-contrast liver and Sector Assist workflow, with automatic atlas application and subsequent manual editing of ROIs in case of deviation of the segmentation lines.

Although the dosimetric parameter used in TARE studies is usually the mean tumor-absorbed dose, we also considered measuring the maximum absorbed dose to the tumor, the minimal absorbed dose in 30, 50 and 70% of the tumor volume, extracted from the standard dose–volume histogram (DVH), and mean absorbed dose in the non-tumor liver, adjacent to the segment or segments treated.

To calculate the actual absorbed dose (aAD), ROIs were performed on the CT image, in most cases contrast-enhanced. The maximum dose, mean dose, minimum dose, and standard deviation were collected from the ROIs.

The final absorbed dose in the lung was determined through the planar scintigraphic image of Tc-99 m-MAA, after calculating the hepatopulmonary fraction and knowing the administered activity, by applying the partition model.

### Follow-up

For the assessment of tumor response, the index tumor was defined as the target tumor treated by radioembolization.

Radiological response control was performed 3 and 6 months after the procedure using multiphase CT, reviewed and reported by liver-dedicated radiologists, with more than 15 years of experience. To assess radiological response, localized mRECIST criteria were used [20], rating the response as complete response (CR), partial response (PR), stable disease (SD), or progressive disease (PD). Finally, the objective response rate (ORR) was defined as a CR or PR. Only the local response of the tumor was assessed, not the rest of the liver disease or extrahepatic disease in case of progression in locations other than the treated tumor.

### Statistical analysis

Statistical data analysis was performed using SPSS Statistic 22.0 software.

The inference was made using a chi-square test for categorical variables and the Student's t test or the Mann–Whitney U test depending on the adjustment to normality.

Initially, a descriptive analysis of the categorical and numerical variables was carried out. The degree of agreement between the theoretical absorbed dose and the actual dose was studied using the Bland–Altman plot, while the correlations with Spearman's Rho. Possible cutoff points of the dosimetric parameters were studied using ROC curves. Finally, bivariate analysis according to the type of microsphere and multivariate analysis were performed.

## Results

### Patient features

Thirty initially included patients underwent radical segmentectomy since March 2020. Six patients were excluded, four due to loss to follow-up at 3 and 6 months, one due to post-treatment residual activity > 20% and one due to death, diagnosed with bilateral pneumonia not attributed to radioembolization therapy (lung mean AD 1.25 Gy). Finally, 24 patients were included diagnosed with HCC CHILD A5 (15), A6 (5) and B7 (4), BCLC stage 0 (5), A (17) and B (2), 19 (79.17%) were men and 5 (20.83%) women, with a mean age of 62.8 years (39 to 80 years). Biochemical liver function parameters collected (bilirubin, albumin, ALT, and AST) and patients features are reflected in Table 1.

The mean yttrium-90 administered activity was 1.38 GBq (SD 1.27). The mean volume to be treated (perfused tissue or angiosome that includes the lesion) was 206.9 cc (SD 164.3) and the mean tumor volume was 56.01 cc (11.33 to 168.17 cc).

For logistical reasons, 87.5% were treated with glass microspheres and 12.5% with resin microspheres (Table 2).

### Dosimetric results

The mean theoretical dose to the tumor (tAD), calculated using MIRD and partition model, depending on whether treatment with glass microspheres or resin, was 306.3 Gy (SD 55.78), while the actual absorbed dose in the tumor, calculated using a voxel-based multicompartmental model (aAD) was 352 (SD 234.6). No significant differences were observed between mean dose values calculated with both methodologies. On the other hand, a very low concordance between dose levels calculated by both methodologies was observed with a correlation coefficient of rho_c 0.027 (considered poor < 0.90). This could be related to the fact that there was important variability in the obtained results of actual absorbed dose calculated by multicompartment model (127.4–1171 Gy) not observed in the conventional calculation models (Figure 3).

The mean maximum tumor dose was 1216 Gy (SD 656.4), with minimal doses measured by dose–volume histogram (DVH) in 30, 50, and 70% of the tumor of 398.9, 303.5, and 238.7 Gy, respectively, while the mean absorbed dose in non-tumor liver was 11.64 Gy (SD 10.17) and in lung 6.88 Gy (SD 7.45).

### Treatment response

**Fig. 1:**
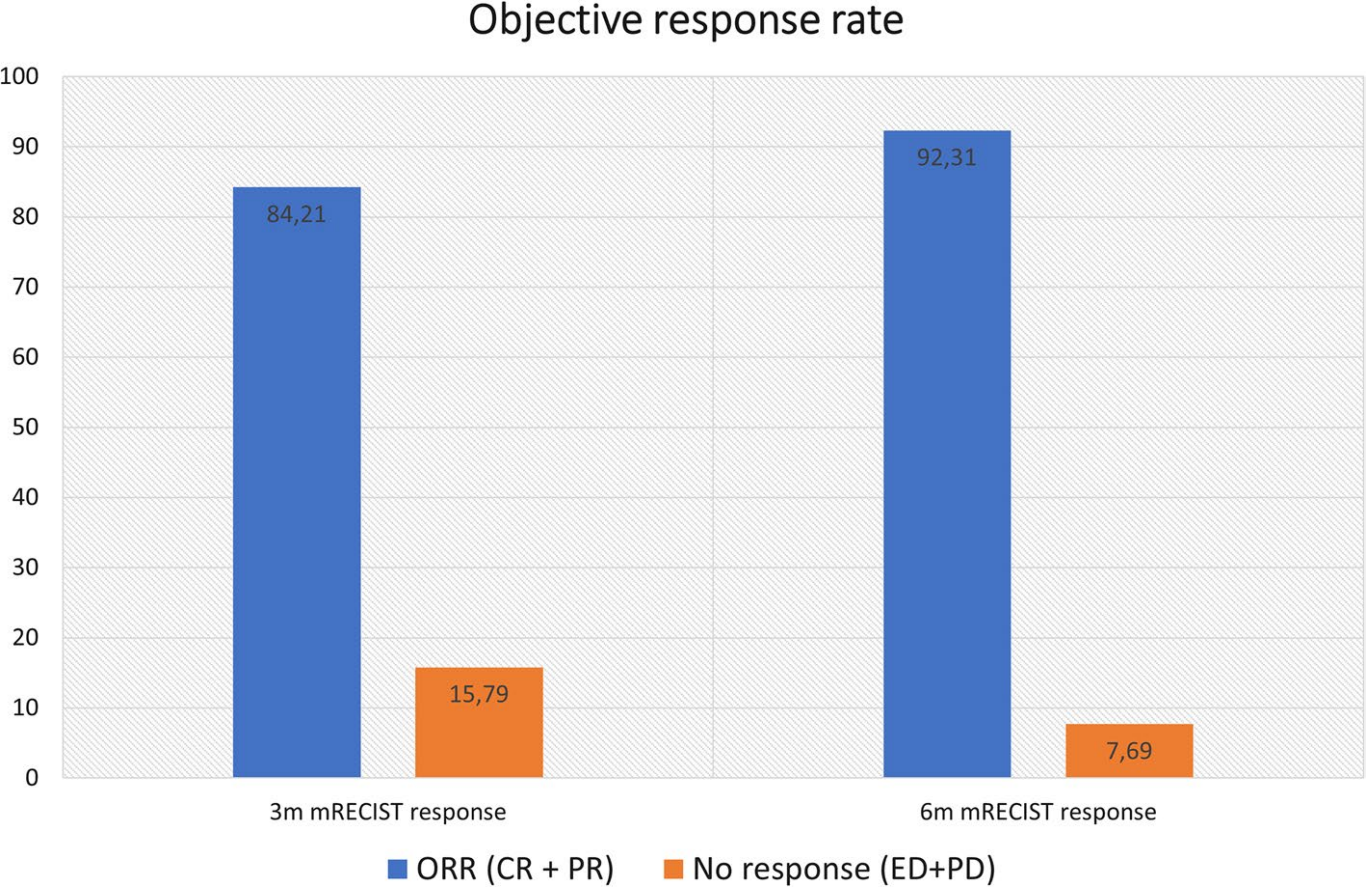
Objective response rate at 3 and 6 months. An evident radiological response, defined as CR or PR, was observed in both intervals, more evident at 6 months

At 3 months, radiological evaluation using localized mRECIST criteria showed ORR (CR, PR) in 84.21%, while ORR was not obtained (SD, PD) in 15.79%. At 6 months, the ORR was achieved in 92.31% (Fig. 1).

**Fig. 2:**
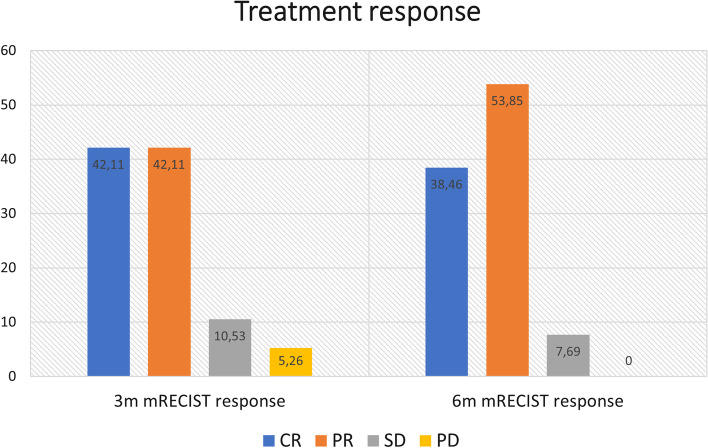
Response stratification using mRECIST criteria at 3 and 6 months

When stratifying response in CR, PR, SD, and PD, 42.11, 42.11, 10.53, and 5.26%, respectively, was obtained at 3 months and 38.46, 53.85, 7.69, and 0%, respectively, at 6 months (Fig. 2).

Bilirubin, albumin, ALT, and AST at 3 and 6 months were 0.97, 3.9, 34.8, 43.2 and 1.03, 3.95, 44.31, 60.27, respectively. Analytical parameters did not change significantly after treatment, as given in Table 3.

**Table 3:** Analytical values evolution

		Range	IQR
Patient (n)	24		
*Baseline analysis*			
Br	0.9	(0.3–4.1)	0.5–1
Alb	4,01	(2.5–4.8)	3.7–4.5
ALT	37.9	(16–82)	25–49
AST	38.3	(20–50)	31–47
*3 m analysis*			
Br	0.97	(0.3–3.6)	0.6–1.25
Alb	3.9	(2.8–4.4)	3.8–4.2
ALT	34.8	(12–72)	24.5–42.5
AST	43.3	(18–80)	29–57
*6 m analysis*			
Br	1.03	(0.4–2.5)	0.7–1.1
Alb	3.9	(3.2–4.6)	3.6–4.4
ALT	44.3	(18–149)	30–45
AST	60.3	(20–214)	40–57

*Br*: Bilirubin; *Alb*: Albumin; *ALT*: alanine aminotransferase; *AST*: aspartate aminotransferase; *IQR*: interquartile range; *m*: months

### Response predictor parameters

By stratifying the response into CR, PR, SD and PD, a statistically significant relationship was detected between the maximum tumor-absorbed dose and complete radiological response at 3 months (p 0.022). Univariate ROC curves of CR at 3 months showed an area under the curve (AUC) of the maximum tumor-absorbed dose of 0.818. A 959 Gy cutoff point with sensitivity (CI95%) 87.5% (47.3–99.7), specificity 72.7% (39.0–94.0), PPV 70.0% (34.8–93.3), and NPV 88.9% (51.8–99.7) (Figure 1).

Univariate ROC curves of ORR at 3 months showed an AUC of 0.708 and a cutoff point of 820 Gy with sensitivity (95% CI) 75.0% (47.6–92.7), specificity 66.7% (9.4–99.2), PPV 92.3% (64.0–99.8) and NPV 33.3% (4.3–77.7).

The correlation analysis also showed, although without statistical significance, a progressive trend in the relationship between CR at 3 months and minimal absorbed dose in 30% of the tumor volume, progressively decreasing such correlation in 50% and 70% of the volume (p 0.22, 0.680 and 0.804, respectively).

At 6 months, results appeared to show a trend toward a correlation between CR and the minimal absorbed dose in 30% of the tumor volume, but did not reach statistical significance (p 0,107).

No differences were observed in terms of radiological response at 3 and 6 months according to the type of microsphere, administered activity (p 0.503 and 0.848, respectively), the tAD to tumor (p 0.419 and 0.959, respectively),or aAD (p 0.495 and 0.853, respectively).

## Discussion

TARE with a supraselective approach, aimed at treating early-stage HCC with minimal tumor load, restricted to one or two segments, can achieve very powerful results in terms of tumor necrosis [21, 22]. Until a few years ago, this procedure was used in patients with a higher tumor burden and in less selective approaches. In addition, the dosimetric analysis of the bulk of the scientific evidence in TARE is based on predictions obtained with the theoretical, MIRD, and compartmental models [16, 23–27]. Our study bases its results on multicompartmental voxel-based dosimetric analysis. This new dosimetric analysis paradigm allows a much more accurate approximation of the absorbed dose in each of the voxels that make up the tumor, especially useful in lesions with mixed irrigation in which the distribution of the particles may be unequal.

In heterogeneous lesions, where solid areas of tumor viability and necrotic or scar tissue from previous treatments coexist, the value of the average absorbed dose is suboptimal, since not all the tumor territories receive the same amount of microspheres and, therefore, of radiation. The same occurs in lesions with mixed irrigation, by more than one nutrient vessel, in which the treatment is performed from more than one arterial feeder. As Yung Hsiang Kao has recently mentioned, tumor-absorbed dose heterogeneity is natural, unavoidable and always present in every patient, without exception [28]. Also Pasciak et al. establish that differences in microsphere-number density may have an effect on microscopic tumor-absorbed dose inhomogeneity [29]. In this sense, voxel dosimetry could more faithfully reflect the heterogeneous distribution of particles, allowing the tumor to be compartmentalized and accurately recognizing the degree of beta radiation deposition in the tumor, offering knowledge of the maximum tumor-absorbed dose to the total of the tumor volume and to the different percentage volumes.

**Fig. 3:**
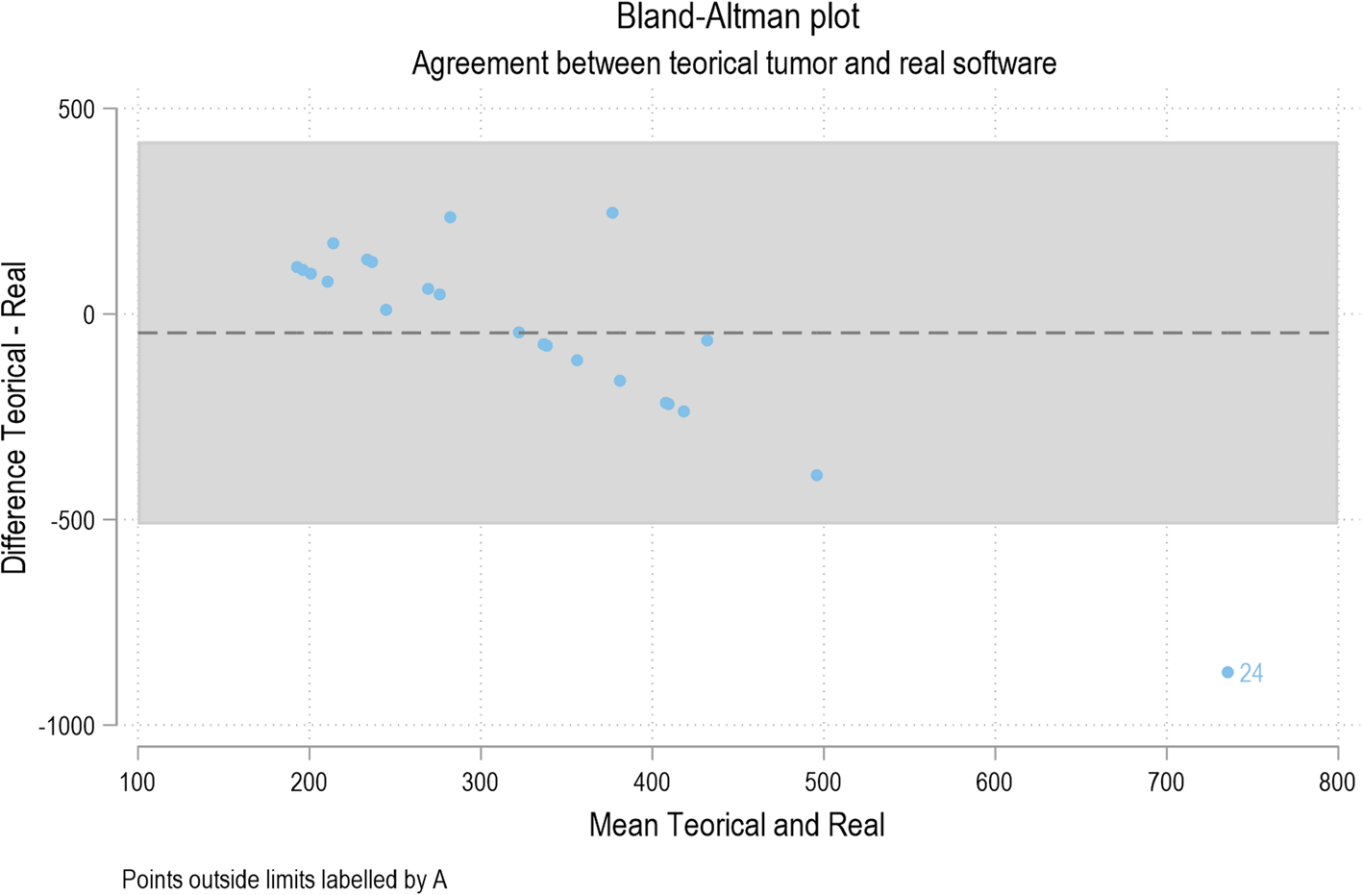
Bland–Altman curve. It reflects that as the absorbed dose values in the tumor increase, the theoretical pretreatment calculation underestimates such dose with respect to the voxel-based dosimetry calculation

**Fig. 4:**
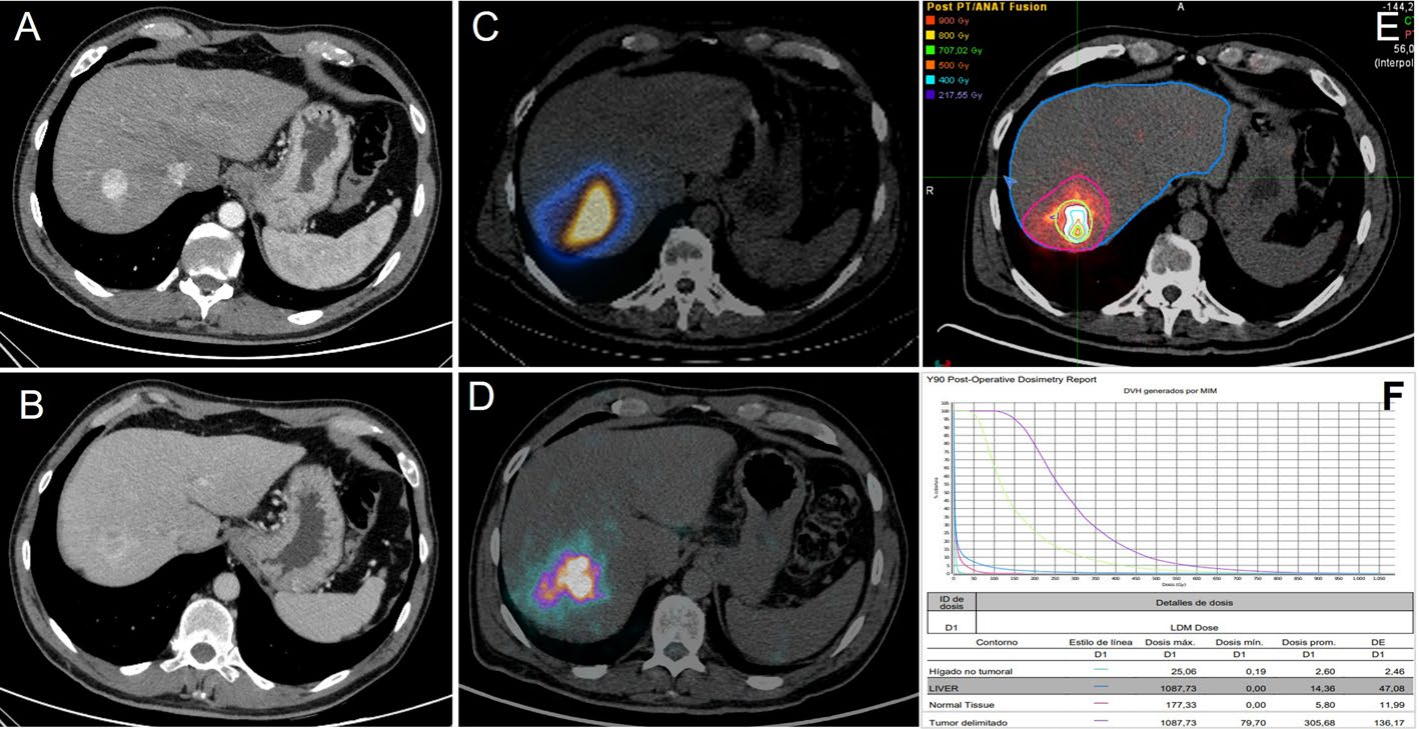
Diagnostic CT in arterial (**A**) and portal (**B**) phases, showing a 23-mm hypervascular nodule in segment VII with contrast washout, diagnosis of HCC. Tc-99 m MAA SPECT/CT (**C**) and Y-90-PET/CT (**D**) image after administration of 0.78 GBq of glass microspheres, showing selective deposition of radioparticles on the tumor and, to a lesser extent, on the rest of the segment. Dosimetric study (**E** and **F**) using MIM software, maximum tumor dose 1087 Gy, mean dose 305 Gy

**Fig. 5:**
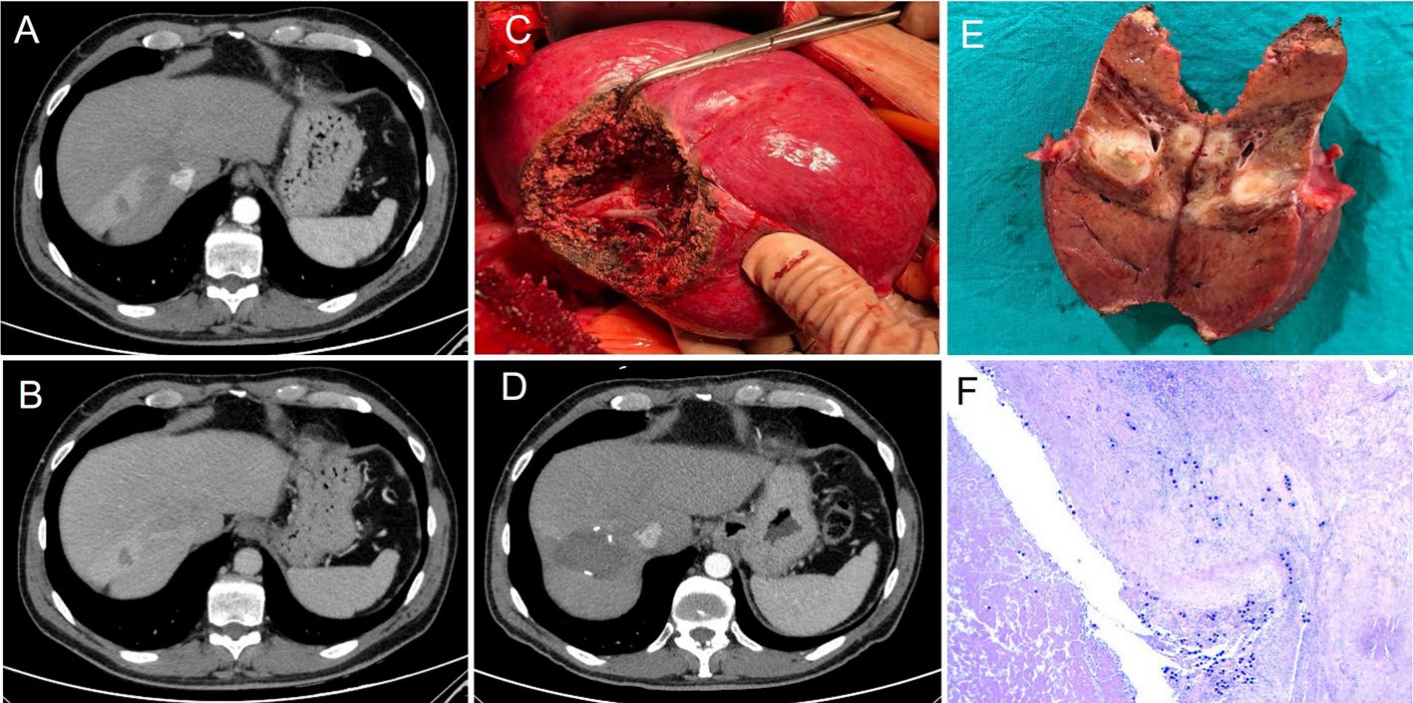
Post-treatment images of the patient diagnosed with HCC (Fig. 4): The CT study to assess response at 3 months (**A** and** B**) showed a 15-mm focal lesion in segment VII without contrast enhancement that could suggest tumor persistence or recurrence. After hepatic segmentectomy (**C** and** D**) performed 9 months after radioembolization, a resection cavity was shown, without focal hepatic lesions suggestive of tumor recurrence. Finally, the pathological study of the surgical piece (**E** and** F**) showed a fibronecrotic nodule in relation to the embolization material without the presence of residual neoplasia

In our study group, even when the tAD to tumor was around 300 Gy, the results obtained using voxel-based dosimetry software showed a striking disagreement, reaching mean Grays values for the tumor between 127.4 and 1171 Gy (Fig. 3). We hypothesize that this great disagreement between the theoretical and actual values may respond to changes in pretreatment volumes (estimated by CBCT) and actual treated tissue volume and/or incomplete administration of the calculated dose which could in turn be explained by possible arterial spasms, flow redistribution, changes in tumor vascularization or technical reasons dependent on minimal variations in catheter placement. The different distribution of MAA in relation to the yttrium microspheres should be taken into account, as well as possible eventualities in vial handling (resin microspheres), delay in treatment, activity decay, among others (Figs. 4, 5).

In this analysis, the dosimetric value with the greatest predictive capacity for response was the maximum tumor-absorbed dose. A maximum dose value greater than 959 Gy was associated with CR at 3 months with a sensitivity and specificity of 87.5% and 72.7%, respectively (p = 0.022), in such a way that 87% of patients receiving a dose above this threshold achieved complete response. Similarly, a maximum dose value of 820 Gy predicted ORR (CR + PR) with a sensitivity and specificity of 75.0% and 66.7%, respectively.

Otherwise, the observation of a greater tendency to complete response according to the minimal dose reached in 30% of the tumor volume (a tendency that progressively decreases in 50 and 70% of the tumor volume), would allow us to hypothesize that rather than the mean absorbed dose to the entire lesion, the parameter that best predicts response is the maximum dose, which translates the maximum concentration of microspheres in the most solid, viable and hypervascularized portion of the lesion and that ultimately is the tissue that determines malignant behavior, evolution and response of the lesion. In any case, radiological response rate was excellent, even in lesions that received less aAD, which suggests that a tAD of 300 Gy calculated using conventional theoretical models would predict clinical benefit at 3 and 6 months in 84.21% and 92.31% of the patients, respectively. These results go hand-in-hand with what has been found in the literature, confirming that radiation segmentectomy offers high radiological response rates [1, 7–9, 12, 14].

In the TARGET study, a recent retrospective multicenter study, the ORR was 70.8 and 61.7% for the target lesion and for all lesions, respectively, assessed by mRECIST, in which responding patients showed a mean tumor-absorbed dose (225.5 Gy) 17% higher than non-responders (188.3 Gy) with a higher OS (36.7 months) in patients with a higher absorbed dose (> 300 Gy)[13]. Similarly, authors of the LEGACY study report a high median perfused volume absorbed dose of 410 Gy with TheraSphere, which resulted in an 88% best response (i.e., CR, PR) by localized mRECIST in patients with early and advanced HCC [12]. These radiological response rates are very similar to those obtained in our study, also coinciding with the sectoral approach to treatment.

In addition, our results, although mostly representative of TheraSpheres because only 12.5% of our patients were treated using SIR spheres, allow us to confirm that the maximum tumor response is obtained between 4 and 6 months after the procedure is performed, as has been described in previous studies [6, 30–32].

We consider this study newfangled since the recommended dosimetric thresholds for liver radioembolization treatments have been obtained based on theoretical models. On the other hand, the therapy approach has changed in recent years toward a greater selectivity. In our study, we have included patients ambispectively, with treatment volumes that do not exceed two liver segments and that have been treated with radical intention. In addition, we have performed the multicompartmental voxel-based dosimetric study of all the radiation segmentectomies. Regarding the use of the two types of microspheres (resin and glass), although different radiobiological effects have been described, noting the different specific activities, number, and size of the spheres and some research papers have explored this, the discussion probably has not arrived any conclusive consensus on these differences or how to still manage them dosimetrically/radiobiologically. That is why we have not applied a conversion factor to determine the equivalent uniform doses.

The main weaknesses of our study are the low sample size and the short follow-up, and we consider it highly relevant to carry out further longitudinal dosimetric studies, with a larger sample volume and with long-term follow-up data.

In conclusion, it is confirmed that a segmental approach directed at lesions not amenable to ablative treatments and calculating treatment doses with radical intention (> 300 Gy), leads to response rates greater than 90%, being the tumor maximum absorbed dose the dosimetric parameter that best predicts radiological response. Even so, we consider it necessary to carry out well-designed prospective studies, with a larger sample size, to establish possible predictive dosimetric thresholds of response and, therefore, benefit in terms of survival.

## Data Availability

All data generated or analyzed during this study are included in this published article [and its supplementary information files].

## Abbreviations

TARE: Transarterial radioembolization
SIRT: Selective internal radiation therapy
HCC: Hepatocellular carcinoma
AD: Absorbed dose
tAD: Theoretical absorbed dose
aAD: Actual absorbed dose
PET: Positron emission tomography
CT: Computed tomography
mRECIST: Modified response evaluation criteria in solid tumors
CBCT: Cone beam CT
BCLC: Barcelona clinic liver cancer
SPECT: Single photon emission tomography
MAA: Macro-aggregates of albumin
TNR: Tumor to nontumoral liver uptake ratio
ORR: Objective response rate
CR: Complete response
PR: Partial response
SD: Stable disease
PD: Progressive disease
Gy: Gray
ROC curve: Receiver operating characteristic curve
AUC: Area under the curve
